# Results of Combined Cyclectomy/Trabeculectomy Procedure Compared with Ahmed Glaucoma Valve Implant in Neovascular Glaucoma Cases

**DOI:** 10.5402/2011/680827

**Published:** 2011-12-08

**Authors:** Kaya N. Engin, Cemil Yılmazlı, Günay Engin, Levent Bilgiç

**Affiliations:** ^1^Department of Ophthalmology, Bagcilar Education and Research Hospital, Bağcılar, 34203 Istanbul, Turkey; ^2^Department of Ophthalmology, Medical Park Bahcelievler Hospital, Istanbul, Turkey; ^3^Department of Ophthalmology, Esenyurt Uğur Hospital, Istanbul, Turkey; ^4^Department of Pathology, Istanbul Medical Faculty, Istanbul University, 34452 Istanbul, Turkey

## Abstract

*Purpose*. Cyclectomy/trabeculectomy and Ahmed glaucoma valve implant are operations suggested for refractory glaucomas. In this study, we have evaluated the outcomes that we observed with these two techniques in neovascular glaucoma patients. *Material and Methods*. Thirty-five eyes with neovascular glaucomas were included in this study. Ahmed Glaucoma valve (group A) was applied to ten eyes, while cyclectomy/trabeculectomy was applied to 25 eyes (group C/T). Vision, intraocular pressure and complications were evaluated at the end of the first week and after 6 and 12 months. *Results*. Vision preservations were 80% and 92%, and success rates in reducing intraocular pressure were 50% and 72% for Ahmed glaucoma valve and cyclectomy/trabeculectomy groups, respectively. None of the differences in complication rates was found to be statistically significant. *Conclusions*. In the surgical management of neovascular glaucoma, cyclectomy/trabeculectomy was shown to be an alternative to Ahmed glaucoma valve operation.

## 1. Introduction

Glaucoma is the main cause of permanent blindness in the world [1]. Trabeculectomy with or without antimetabolites remains the golden standard for most of the primary and secondary glaucomas when medical and laser therapies are insufficient. However, some eyes are unresponsive to this therapy as well. Neovascular glaucoma (NVG) is reported to be the worst type when it comes to failure rates [2]. Recommendations for treatment of NVG include treatment of the underlying disease, complete panretinal photocoagulation if retinal ischemia is a factor [3], and anterior retinal cryoablation (ARC), especially in eyes with media opacities and as a preliminary procedure for filtering surgery or drainage implant surgery [4]. Although the ideal surgical procedure is still controversial, currently, trabeculectomy with antimetabolite therapy, aqueous shunt implants [5], and diode laser cyclophotocoagulation [6] are the favored surgical treatment options.

Aside from diode laser cyclophotocoagulation, ciliary body has been a focus of interest since Sautter's operation in the early 80′s [7]. Current techniques targeting ciliary body include contact [8] and noncontact transscleral neodynium : yttrium aluminium garnet (Nd : YAG) cyclophotocoagulation [9] and are also useful in refractory glaucomas.

Cyclectomy/trabeculectomy is a modified trabeculectomy operation particularly suggested for refractory glaucomas [10]. Ahmed glaucoma valve surgery, on the other hand, is a well-known glaucoma drainage implant operation for this patient group [11]. NVG has the lowest success rates with conventional and glaucoma drainage implant operations among refractory glaucomas. In this study, we have retrospectively evaluated the patient outcomes that we have observed with these two techniques in patients with NVG.

## 2. Material and Methods

Thirty-five eyes of 33 patients with NVG were included in this study. All the patients had been receiving triple medication consisting of timolol, dorzolamide, and bimatoprost, and they were also under cure in our retina department. Females and males of this group were 14 and 9, respectively, and the average age was 62,7 years (range of 54–79 years). Mean intraocular pressures (IOP) in A and Group C/T were 50,2 and 52,7, respectively. The main etiology was proliferative diabetic retinopathy in both groups. Written approval had been obtained from each patient, and the tenets of the Declaration of Helsinki were adhered to. Ten eyes of 10 patients—5 females and 5 males—were treated with Ahmed glaucoma valve (Group A). For the 25 eyes of 23 cases, cyclectomy/trabeculectomy was undertaken (group C/T). Sole criterion for determining which surgical therapy was to be used for any given patient was his/her preference, following full explanation of the features of both procedures. The main etiology was proliferative diabetic retinopathy in both groups.

Operations were performed under regional anesthesia. Heads of the patients were kept slightly elevated, and they were monitorized to secure the safe and hypotensive surgery. In Group A, after the preparation of fornix-based conjunctival flap, implantation of Ahmed glaucoma valve model S-2 (New World Medical Inc., Rancho Cucamonga, Calif, USA) was performed in superior temporal region—10 mms away from limbus. Before placement of the tube implant body onto the sclera, the tube was irrigated with saline solution to open the valve mechanism. The tube was then trimmed, and the anterior chamber was entered from 1 mm posterior to the corneoscleral limbus with a 23-gauge needle. After the preparation of a half-thickness scleral flap, the tip of the tube was placed leaving 3-4 mm in the anterior chamber. The polypropylene body of the implant was sutured onto the sclera with 8.0 polyester suture.

The tube was covered with the scleral flap with a 10.0 absorbable suture. The conjunctiva was then sutured with a 8.0 polyester.

**Figure 1:**
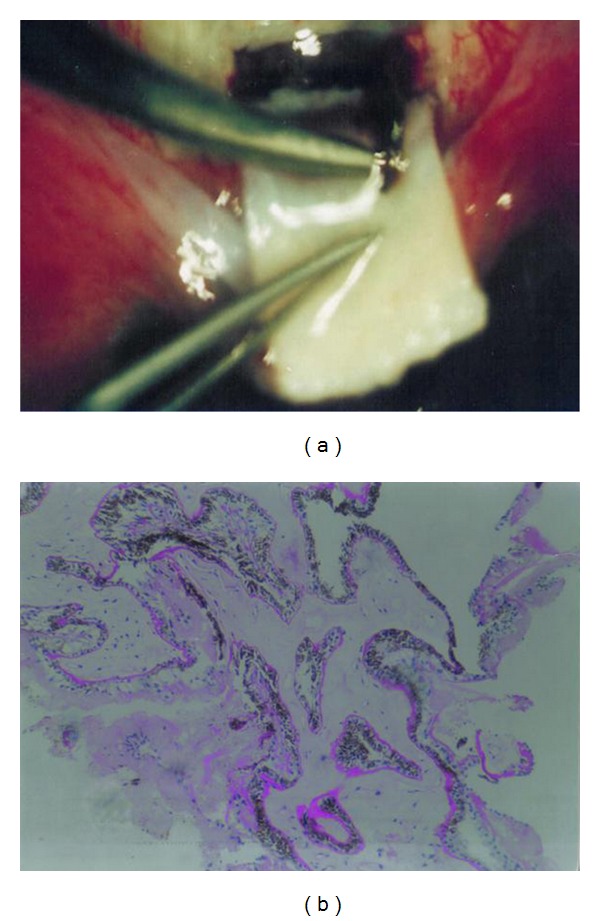
(a) Excision of the ciliary body fragment in the same dimensions of 2 × 4 mm, between the 1 and 3 mm behind the limbus. (b) Pars plicata of the excised ciliary body (H.E. ×100). Pathological view of excised ciliary body fragment.

Cyclectomy/trabeculectomy was applied, as described by Engin G [10, 12]. A limbus-based conjunctival flap was dissected. A half thickness scleral flap (4 × 6 mm) was prepared. In order to prevent potential bleeding, whole deep sclera is cauterized until a mild color change is achieved. This practice also decreases flap adhesion. After excision of a deep scleral piece of 1 to 3 mm, posterior to the trabecular projection on the limbus gray line, a full-thickness ciliary body fragment of the same dimensions of 2 × 4 mm was excised between the 1 and 3 mm posterior to the clinical limbus (Figure 1(a)). All the specimens were confirmed to be ciliary body (Figure 1(b)). The anterior chamber was entered just from the bottom of the cornea by separating the cornea from iridocorneal angle with a spatula. After the excision of the trabeculum of 2 × 3 mm with peripheral iridectomy, the operation was completed with closing of the scleral flap with 2 or 3 10.0 absorbable sutures and closing of the conjunctival flap by a 8.0 polyester running suture. No extra surgical device was used for the C/T operation, and no antimetabolites were used in either group. Age, gender, visual acuity, and IOP were documented for each patient preoperatively. Postoperatively topical corticosteroids (dexamethasone 0,1% 6 times/day), antibiotics (tobramycin 0,3% 6 times/day), and cycloplegic drops (tropicamide 1% 3 times/day) were administered to all patients for one month. Prostaglandin analogues and/or aqueous suppressants were added to the therapy in eyes bearing IOP higher than 21 mmHg or complication. Postoperative comfort was evaluated with questionnaires regarding complaints such as pain, photophobia, and ocular irritation. Data regarding postoperative followups were analyzed retrospectively. Best corrected visual acuities with Snellen charts, IOPs taken by applanation tonometer and complications determined in routine exminations were recorded at the end of the first week and after 6 and 12 months. Cases with IOP between 5 to 20 mmHg, without additional medical therapy, were considered as a complete success in the means of IOP lowering, whereas IOP less than 5 mmHg was recorded as hypotony. The data is presented as the mean ± standard deviation. Nonparametric Mann-Whitney *U* test was used to indicate and compare IOP reductions in both groups. Success and complication rates and vision preservations were compared with Fisher's exact test. Differences were considered significant with a *P* < 0.05 to reject the null hypothesis

## 3. Results

**Figure 2:**
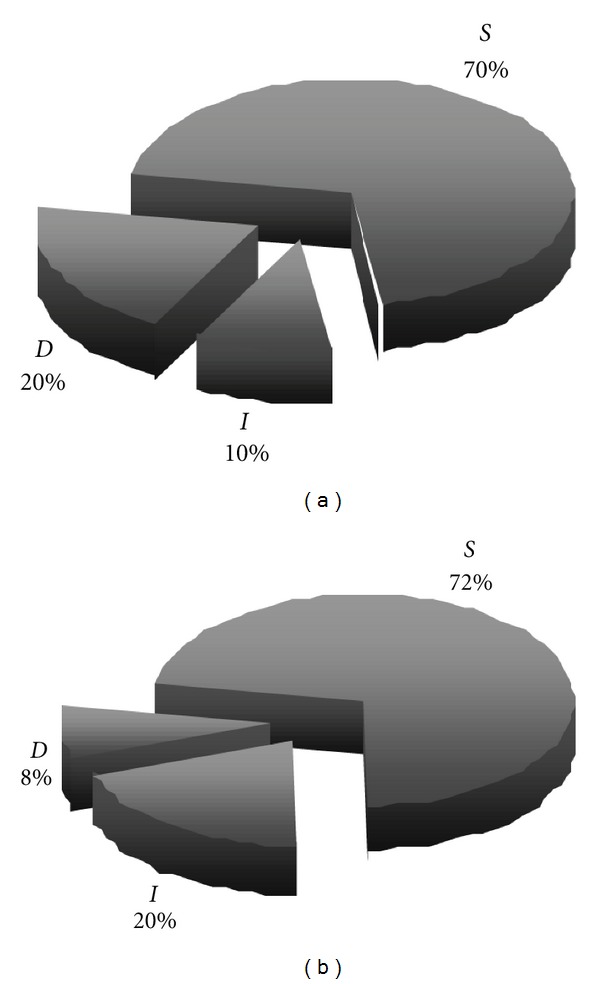
Comparisons of postoperative distribution of visual outcome in Group A (a) and Group C/T (b) patients. D: decreased, I: increased, S: same.

**Figure 3:**
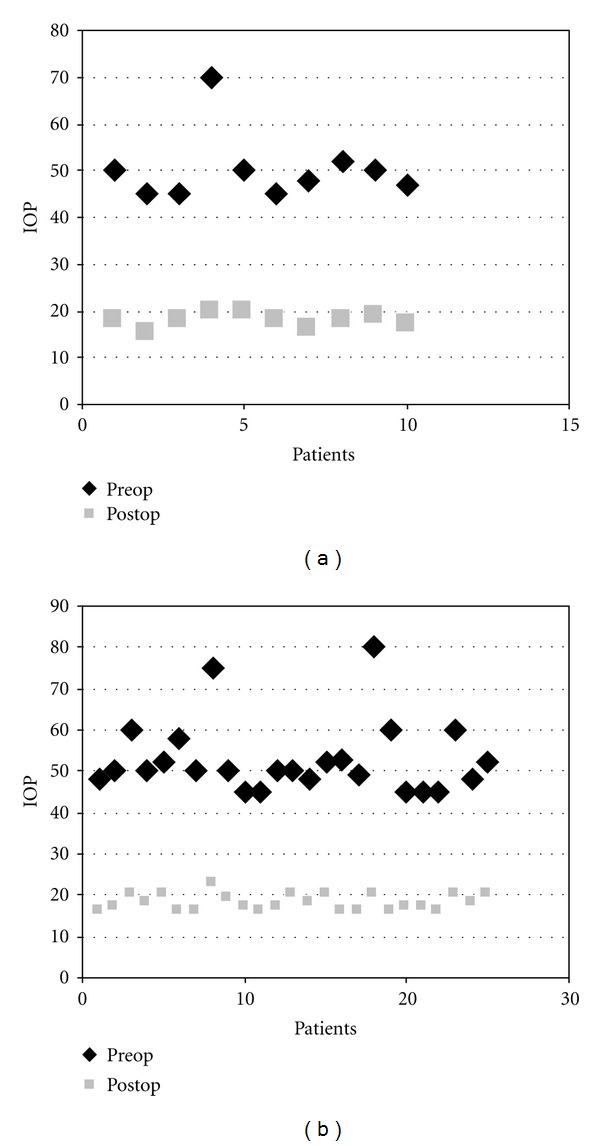
Comparisons of pre- and postoperative intraocular pressures in A (a) and C/T (b) groups. Preop: Preopaerative, Postop: postoperative, IOP: intraoculer pressures.

At the end of the 1st year, vision preservations were found to be 80% and 92% in A and Group C/T (Figure 2), respectively. This difference was not statistically significant (*P* = 0,5607). Mean intraocular pressures significantly (*P* > 0,0001) reduced in both groups after the operations (Figure 3) (Table 1), and there was no significant difference between the follow-up IOP values of the two groups. Although the success rates were 50% and 72% for A and Group C/T, respectively, that data was also not statistically significant (*P* = 0,2577). Complaints such as photophobia and ocular irritation disappeared in all patients, and postoperative patient comfort was barely better in Group C/T than Group A.

Regarding complications (Table 2), corneal decompensation, cystic bleb, and enucleation (10% each) were seen in Ahmed glaucoma valve group. On the other hand, transient hyphema, which resolved spontaneously within 2–5 days, was more frequent in Group C/T than that in Group A (40% versus 10%). Hemorrhages originated from the iris base (not from ciliary body). They were not abundant and stopped spontaneously. More protracted bleedings were observed in 3 eyes but they were easily controlled by cauterization. No shallow anterior chamber after the operation was observed despite the occurrence of choroidal detachment (12%). Serous retinal detachments that we observed (4%) were limited to the inferior part of the retina. It sustained for 2 weeks and then resolved spontaneously without any effect on the visual acuity. No zonular damage and subluxation of the lens were observed; however, in 2 cases, lens touch precipitated cataract formation (8%). None of the differences in complication rates were found to be statistically significant. Perioperative problems in both groups were quite rare. Vitreous loss was observed in a few patients in which anterior vitreous and hyaloid integrity were broken. A small vitreous excision was performed in order to prevent a blockage at the filtration site.

## 4. Discussion

Despite the recent advances in ophthalmic surgery, lasers, and molecular biology, refractory glaucomas, especially NVG, remain a huge problem for ophthalmologists, and surgery is still the last resort in these cases [3]. Other alternatives including antimetabolites [11–13], anterior retinal cryoablation [4], or antivascular agents [14] are used with or without surgical methods. Retinal applications targeting underlying causes are other options that must be considered [15].

**Figure 4:**
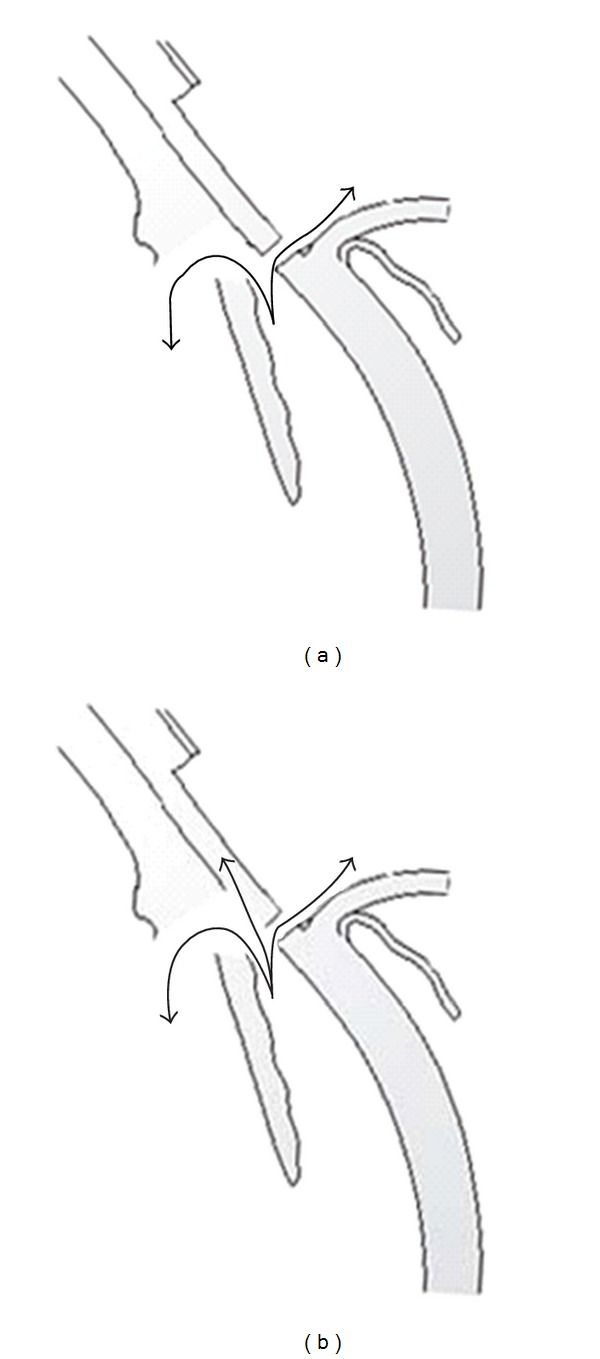
Larger aqueous outflow compared with standard trabeculectomy (a), by several ways including directly subconjonktival, subchoroidal, and probably intravitreal (b).

Aqueous shunts are useful options in the management of complicated glaucoma, where conventional filtration surgery is considered to carry a high risk of failure. Ahmed glaucoma valve is an accepted device that has integrated mechanisms to sustain a residual intraocular pressure in order to avoid postoperative hypotonia and related complications [16]. According to a comparative retrospective study reported by Taglia et al., success rates—IOP below 15 mmHg at 1 year—were 80% for the Molteno implant, 39% for the Krupin eye valve with disc, and 35% for the Ahmed glaucoma valve. However, Ahmed glaucoma valve patients were less likely to experience complications requiring reoperations or loss of two or more lines of visual acuity than the others [11]. In one of the retrospective studies regarding the Ahmed glaucoma valve, postoperatively 15% of 159 eyes were reported to have intraocular pressure equal or greater than 22 mmHg. According to that study, the complication rate was 47% and the most common complication was obstruction of the tube (11%) [17]. In a study performed specifically on NVG patients, cumulative probabilities of success were 63.2% after one year in Ahmed glaucoma valve group [5]. Although the evaluation criteria were different, our 50% success rate in Group A was poorer than this study, but higher than that of Taglia et al. [11]. A surgical technique on the ciliary body to reduce aqueous formation by excising partially the pars plicata was described by Sautter in 1980s [5]. Both success rate and vision preservation were reported as 60/85. When compared with the Sautter operation in which 60°–150° of the pars plicata was excised, the filtering effect of our technique is more important than the decrease of aqueous formation (Figure 4) [10, 12]. That difference also made us avoid their major haemorrhage complication even in cases with neovascular glaucoma. A more recent technique aiming at the ciliary body is the cyclophotocoagulation. A long-term followup of 500 patients treated with noncontact transscleral Nd : YAG cyclophotocoagulation was carried out by Shields et al. Satisfactory intraocular pressure reduction was achieved in 62% and 87% of the patients with single and repeated treatment sessions, respectively.

However, visual loss remained a significant postoperative complication, with some degree of reduced vision occurring in 39% of the study population. Patients with neovascular glaucoma yielded the greatest rate of visual loss at 46% [8]. Unsatisfactory intraocular pressure reduction and reduced vision rates for contact transscleral cyclophotocoagulation were reported as 50% and 27%, respectively [9]. Vision preservations were found to be 80% and 92% in A and Group C/T, respectively, which were both higher than 76.4% of Yalvac et al. in NVG [5]. Visual acuity reduced in two eyes due to cataract formation in Group C/T, and this was not a specific complication of the cyclectomy/trabeculectomy operation [18]. In Group A, however, loss of light perception was observed in one patient. The loss of vision secondary to the progression of underlying disease in neovascular patients was encountered in 22.2% of the patients following Ahmed glaucoma valve implantation [5]. In literature, loss of light perception in NVG was encountered as 31% and 48% after Baerveldt [19] and Molteno [20] tube implantations, respectively.

The most common complication seen in the group C/T was hyphema. In a study reporting results after Trabeculectomy with MMC combined with direct cauterization of peripheral iris in patients with NVG, 20.8% hyphema was reported, in which irrigation of anterior chamber was required for 3 eyes [13]. We had a rate of 40% but all the hyphemas were resolved spontaneously in our series. That rate was also higher than 31% of our previous series with various aetiologies [10]. Consistent with the findings of Yalvac et al., transient hyphema was also the most common complication of the Ahmed glaucoma valve group in our series, with an occurrence of 18.4% versus 10% of the Group C/T [5]. It was reported between 8 and 20% with tube implantation in NVG [19, 20]. Hypotony and corneal decompensations were found to be 10% in our series, while the occurrence of these complications were both reported previously as 5,3% [5]. This may be attributed to the small size of our series with Ahmed valve. Postoperative hypotony was reported between 8 and 13% in other Ahmed glaucoma valve series [21, 22]. No corneal decompensation was observed in Group C/T but hypotony was found to be 26%. Although that rate is higher than those previously reported in the literature, it was not a major problem clinically.

When we compare C/T procedure not only with Ahmed glaucoma valve operation, but with the other well-known techniques above, as far as IOP reducing effect and vision preserving properties are concerned, the superiority of C/T technique is clearly seen. Clinical trials comparing C/T technique with standard trabeculectomy, however, would be of great importance. On the other hand, the complications that we observed were less frequent and not worse than standard trabeculectomy in incidence and severity [2]. Other advantages of C/T technique over other alternatives are the ease of adaptation for a surgeon who is used to performing classical trabeculectomy and the occurrence of large aqueous outflow by several routes including suprachoroidal and probably intravitreal. The results show that, when surgery is the management of choice, C/T is a favorable and cost-effective option in the management of neovascular glaucoma.

## Figures and Tables

**Table 1:** Mean IOP profile of Group A and C/T patients before and after surgery.

	Preop.	1st week	6st month	12st month
Group A	50,2 ± 7,40	17,9 ± 1,59	20,5 ± 1,27	21,5 ± 2,45
Group C/T	52,8 ± 8,60	17,9 ± 1,95	19,7 ± 1,51	21,2 ± 1,7

**Table 2:** The rate and percentages of postoperative complications in Group A and C/T.

	Group A	Group C/T
Hyphema	1/10 (10%)	10/25 (40%)
Anterior chamber inflammation	2/10 (20%)	4/25 (16%)
Hypotony	2/10 (20%)	6/25 (26%)
Choroidal detachment	1/10 (10%)	3/25 (21%)
Intravitreal hemorrhage	None	2/25 (7%)
Serous retinal detachment	None	1/25 (5%)
Secondary cataract	None	1/25 (5%)
Shallow anterior chamber	3/10 (30%)	4/25 (16%)
Corneal decompensation	1/10 (10%)	None
Cystic bleb	1/10 (10%)	None
Enucleation	1/10 (10%)	None

## References

[1] Coleman AL, Brigatti L (2001). The glaucomas. *Minerva Medica*.

[2] Mietz H, Raschka B, Krieglstein GK (1999). Risk factors for failures of trabeculectomies performed without antimetabolites. *British Journal of Ophthalmology*.

[3] Sivak-Callcott JA, O’Day DM, Gass JD, Tsai JC (2001). Evidence-based recommendations for the diagnosis and treatment of neovascular glaucoma. *Ophthalmology*.

[4] Sandramouli S, Sihota R, Sood NN (1993). Role of anterior retinal cryoablation in the management of neovascular glaucoma. *Documenta Ophthalmologica*.

[5] Yalvac IS, Eksioglu U, Satana B, Duman S (2007). Long-term results of Ahmed glaucoma valve and Molteno implant in neovascular glaucoma. *Eye*.

[6] Pokroy R, Greenwald Y, Pollack A, Bukelman A, Zalish M (2008). Visual loss after transscleral diode laser cyclophotocoagulation for primary open-angle and neovascular glaucoma. *Ophthalmic Surgery Lasers and Imaging*.

[7] Sautter H, Demeler U (1984). Antiglaucomatous ciliary body excision. *American Journal of Ophthalmology*.

[8] Shields MB, Shields SE, Kass MA, Stark W, Taylor H (1994). Noncontact transscleral ND:YAG cyclophotocoagulation: a long-term follow-up of 500 patients. *Transactions of the American Ophthalmological Society*.

[9] Seah SK, Jap A, Min G (1994). Contact transscleral cyclophotocoagulation for end stage glaucoma. *Annals of the Academy of Medicine Singapore*.

[10] Engin G, Yilmazli C, Engin KN, Gülkilik G, Bilgic L (2004). Combined cyclectomy-trabeculectomy procedure for refractory glaucoma. *Ophthalmic Surgery Lasers and Imaging*.

[11] Taglia DP, Perkins TW, Gangnon R, Heatley GA, Kaufman PL (2002). Comparison of the Ahmed Glaucoma Valve, the Krupin Eye Valve with Disk, and the double-plate Molteno implant. *Journal of Glaucoma*.

[12] Engin G, Engin KN, Bilgiç L (2006). A modified surgical method for difficult glaucomas. *Techniques in Ophthalmology*.

[13] Elgin U, Berker N, Batman A, Simsek T, Cankaya B (2006). Trabeculectomy with mitomycin C combined with direct cauterization of peripheral iris in the management of neovascular glaucoma. *Journal of Glaucoma*.

[14] Cheng JYC, Wong DWK, Chong LA (2008). Intraocular avastin (bevacizumab) for neovascularisation of the iris and neovascular glaucoma. *Annals of the Academy of Medicine Singapore*.

[15] Kiuchi Y, Nakae K, Saito Y, Ito S, Ito N (2006). Pars plana vitrectomy and panretinal photocoagulation combined with trabeculectomy for successful treatment of neovascular glaucoma. *Graefe’s Archive for Clinical and Experimental Ophthalmology*.

[16] Hille K, Moustafa B, Hille A, Ruprecht KW (2004). Drainage devices in glaucoma surgery. *Klinika Oczna*.

[17] Huang MC, Netland PA, Coleman AL, Siegner SW, Moster MR, Hill RA (1999). Intermediate-term clinical experience with the Ahmed Glaucoma Valve implant. *American Journal of Ophthalmology*.

[18] Mermoud A, Salmon JF, Alexander P, Straker C, Murray ADN (1993). Molteno tube implantation for neovascular glaucoma: long-term results and factors influencing the outcome. *Ophthalmology*.

[19] Sidoti PA, Dunphy TR, Baerveldt G (1995). Experience with the Baerveldt glaucoma implant in treating neovascular glaucoma. *Ophthalmology*.

[20] Hylton C, Congdon N, Friedman D (2003). Cataract after glaucoma filtration surgery. *American Journal of Ophthalmology*.

[21] Huang MC, Netland PA, Coleman AL, Siegner SW, Moster MR, Hill RA (1999). Intermediate-term clinical experience with the Ahmed Glaucoma Valve implant. *American Journal of Ophthalmology*.

[22] Coleman AL, Hill R, Wilson MR (1995). Initial clinical experience with the Ahmed Glaucoma Valve implant. *American Journal of Ophthalmology*.

